# Relationship between insomnia and working from home among Korean domestic workers: results from the 5th Korean working condition survey

**DOI:** 10.1186/s12889-023-16268-5

**Published:** 2023-07-17

**Authors:** Lei Lee, Ok Hyung Nam, Ko Eun Lee, Chunui Lee

**Affiliations:** 1 Department of Internal Medicine, Yonsei University College of Medicine, Seoul, 03722 Korea; 2 Department of Pediatric Dentistry, School of Dentistry, Kyung Hee University, Seoul, 02447 Korea; 3 Department of Pediatric Dentistry, Kyung Hee University College of Dentistry, Kyung Hee University Medical Center, Seoul, 02447 Korea; 4 Department of Oral and Maxillofacial Surgery, Wonju College of Medicine, Yonsei University, Wonju, 26426 Korea

**Keywords:** Mental health, Occupational health, Sleep disorder, Social distancing

## Abstract

**Background:**

Social distancing has been increasingly implemented following the COVID-19 pandemic and more people have been working from home. Consequently, the screen time has increased, which can disrupt the natural sleep-wake cycle and delay sleep onset. Given that studies on the health of employees who work from home remain insufficient, particularly with respect to the risk of sleep disorders including insomnia, this study aimed to assess the relationship between working from home and insomnia among workers using data from the 5th Working Conditions Survey conducted in Korea.

**Methods:**

Of 30,108 wage workers, we enrolled 818 employees who worked from home and 4,090 employees who worked from the office, a 1:5 pair sample based on sex and occupational group. Personal and occupational characteristics, working from home, and insomnia were included in the analysis. Age, education, employment status, working years, working hours per week, work-life balance, self-perceived health, depression, and anxiety were all adjusted as potential confounding variables. Conditional logistic regression analysis was performed using working from home as an independent variable and insomnia as a dependent variable to determine the correlation between working from home and insomnia.

**Results:**

Working from home was associated with sleep onset latency disorder, OR = 3.23 (95% CI: 2.67–3.91), sleep maintenance disorder, OR = 3.67 (95% CI: 3.02–4.45), and non-restorative sleep, OR = 3.01 (95% CI: 2.46–3.67); working from home had a statistically significant relationship with all three types of insomnia.

**Conclusions:**

Within the limits of the study, these findings can be used as a fundamental basis for the implementation of policies and guidelines to prevent insomnia in workers who work from home.

**Supplementary Information:**

The online version contains supplementary material available at 10.1186/s12889-023-16268-5.

## Background

Advancements in information and communication technology, including smart mobile devices and internet-based technology, as well as the popularization of stable wireless connections have expanded the workplace. This has enabled working from home, remote work, and flexible work [1]. For instance, in the United States, the proportion of people working from home increased from 9% to 1995 to 37% in 2015 [2]. A 2018 European report stated that 5.2% of workers worked from home [3]. The COVID-19 pandemic led to the implementation of social distancing and self-isolation regulations, and working from home became an important safety measure [4].

Working from home has several advantages including reducing the cost and time spent commuting to work, personalized work scheduling [5], and improving work-life balance [6]. From a social perspective, it also reduces traffic congestion, eliminates environmental pollution, and promotes productivity. In contrast, as the working and living space is not separated, working from home can result in time mismanagement and lower productivity. The challenges that remote workers must overcome include social isolation, a lack of cooperation and communication among colleagues, and difficulty sending important documents securely and utilizing appropriate technologies. Furthermore, being at home for an extended period can increase the risk of obesity, and the lack of fresh air can contribute to increased tension and anxiety [7].

Adequate sleep is crucial for maintaining physical and mental health. Sleep quality is associated with cardiometabolic factors, such as obesity, diabetes, high blood pressure, and metabolic syndrome [8] as well as mental health factors, such as higher stress, depression, and anxiety [9]. According to data from the Korean National Health Insurance Corporation (2018), approximately 570,000 patients were treated for sleep disorders, accounting for 1.1% of the total population [10].

An irregular schedule due to working from home can lead to the disruption of the sleep-wake cycle, resulting in sleep conditions such as insomnia and circadian rhythm disorders [11], directly affecting sleep. In addition, as physical exercise and outdoor activities promote good sleep quality [12], the sedentary behavior adopted from working from home may negatively affect sleep. The trend toward working from home has resulted in increased screen time [13], and this elevated exposure to blue light emitted by devices can disrupt the natural sleep-wake cycle and delay sleep onset.

The bulk of the research on working from home focuses on productivity, work efficiency, subjective satisfaction, and work-life balance; thus, studies on the health of employees who work from home remain insufficient, particularly those evaluating the risk of sleep disorders including insomnia. Therefore, this study aimed to determine the relationship between working from home and insomnia among Korean workers. Our study results provide valuable information regarding the effects of the increased rate of working from home on workers.

## Methods

### Study population and outline of study protocol

This cross-sectional study used data from the 5th Korean Working Conditions Survey (KWCS) conducted in 2017 by the Institute for Occupational Safety and Health in Korea (https://oshri.kosha.or.kr/oshri/researchField/workingEnvironmentSurvey.do). The Working Environment Survey comprises one-on-one household interviews with employed people aged ≥ 15 years at the national level with various outcomes of interest. The 5th KWCS included a total of 50,205 people over 18.5 weeks from July 11 to November 17, 2017. The KWCS provides open access to nationally approved statistical data with safeguards to ensure participant anonymity and privacy (approval number: 380,002) [14].

**Fig. 1:**
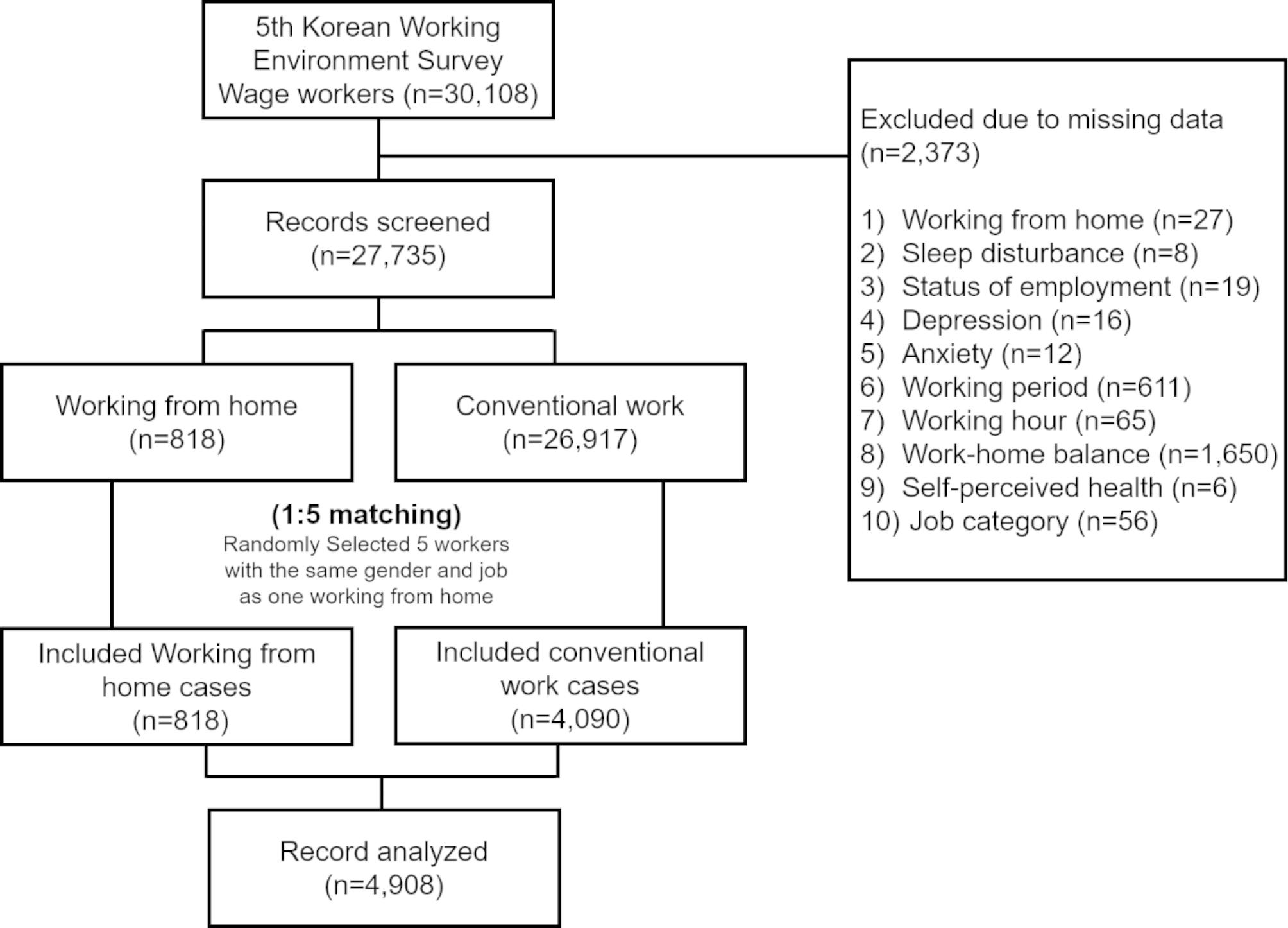
Flow diagram of the inclusion criteria

This study included economically active Korean employees, self-employed workers, and employers who responded to the 5th KWCS. We excluded people with missing data. Of 30,108 wage workers, 2,373 with at least 1 missing value were excluded, and the remaining 27,735 participants consisted of 818 who worked from home and 26,917 who worked from the office. The ratio of those who work from home to those who work from the office was 1:5 and grouping was based on sex and occupation. Ultimately, 4,090 people were enrolled in the conventionalgroup (Fig. 1).

The occupation was categorized into 11 groups according to participants’ responses based on the question, “Which of the following job classifications is most appropriate for your job (occupation)?” Response options were; 1) Manager, 2) Expert, 3) Technician and paraprofessional, 4) Office worker, 5) Service worker, 6) Sales worker, 7) Agriculture, forestry, fishery worker, 8) Technician and related technical worker, 9) Equipment/machine operation and assembly worker, 10) Simple labor worker, and 11) Soldier.”

### Independent variable: working from home

We determined the work-from-home group using the following criteria: (1) people who responded “My house” to the question regarding their main place of work and (2) people who chose “1) Daily,” “2) Several times a week,” and “3) Several times a month” to the sub-question “In the past 12 months, how often did you work primarily in the places?”.

In the past, the term “working from home” was often used interchangeably with “remote work” and “flexible work.” However, the strict meaning is different for all three, as flexible work refers to work with flexible working hours, place, quantity, and continuity, such as in remote work and working from home. Remote work, however, refers to a system in which people work from an office close to their place of residence, business trip, or work using a mobile device at a place other than the office [15]. Working from home involves the use of information and communication devices to prepare a workspace at one’s place of residence rather than the workplace [16]. Previous studies have typically defined those working from home for more than a day per week as workers “working from home” [17].

### Dependent variable: sleep disturbance

The 5th KWCS assessed the prevalence of sleep disturbances using the following three sub-items: “Over the last 12 months, how often did you have any of the following sleep-related problems?” For “difficulty initiating sleep” the response options were “1) Daily” and “2) Several times a week” in response to “A. Difficulty falling asleep”, “1) Daily,” “2) Several times a week,” “3) Several times a month,” “4) Rarely,” and “5) Never.” Responses to “Difficulty maintaining sleep” were “1) Daily” and “2) Several times a week” in response to “B. Waking up repeatedly during the sleeping.“ “Non-restorative sleep” was defined as “1) Daily” and “2) Several times a week” in response to “C. Waking up with a feeling of exhaustion and fatigue.” Sleep disturbance was defined according to the diagnostic criteria A and C from the DSM-V’s definition of sleep disorder and insomnia (ICD disease code G47.00) (S1).

### Confounding variable

In terms of personal characteristics, the educational background was dichotomized as “Below high school graduation” and “College graduate or higher,” and employment status was categorized as “Full-time” or “Part-time.” Working years and working hours per week were analyzed as continuous variables. Work-life balance and self-perceived health were categorized as “3 (good),” “2 (average),” and “1 (poor).” Depression and anxiety were dichotomized into “yes” and “no.”

### Statistical analysis

The chi-square test was used to evaluate the distribution of personal and professional characteristics based on the participants’ place of work. A chi-square test (categorical variables) or a t-test (continuous variables) was performed to assess between-group differences among participants who work from home and those who do conventional work. Categorical variables are expressed as the number of individuals and percentages, whereas continuous variables are presented as means and standard deviations.

Conditional logistic regression analysis was performed to evaluate the association between working from home and sleep disturbances, adjusted for the following covariates: age, education, employment status, working years, working hours per week, work-life balance, self-perceived health, depression, and anxiety. The covariates were selected based on previous studies and were assumed to affect both independent and dependent variables [18]. As we matched participants based on sex and occupation, they were excluded from the covariate sets. All analyses were conducted using SAS (version 9.4; SAS Institute, Inc., Cary, NC, USA), and *P* < 0.05 was considered statistically significant.

## Results

Table 1 shows the personal and occupational characteristics of the included workers. The average age of participants in the working-from-home group was higher than that of the conventional-work group (47.8 ± 14.1 vs. 46.2 ± 13.9 years; *P* = 0.002). Regarding employment status, the proportion of part-time workers was significantly higher in the working-from-home group than in the conventional-work group (23.6% and 19.0%, respectively; *P* = 0.002). The working period (years) and working hours (hours/week) of the working-from-home group were significantly shorter than those of the conventional-work group (*P* = 0.031 and *P* < 0.001, respectively). For the work-life balance, scores tended to be lower for the working-from-home group, suggesting a worse work-life balance than the conventional-work group (*P* < 0.001). The rates of experiencing depression and anxiety in the working-from-home group were significantly higher than those in the conventional-work group (*P* < 0.001). Compared to the conventional-work group, the working-from-home group had significantly more difficulty initiating (39.7% vs. 12.3%, *P* < 0.001) and maintaining (39.2% vs. 10.9%, *P* < 0.001) sleep, and they had more non-restorative sleep (38.3% vs. 13.0%, *P* < 0.001). The proportion of people who experienced all three types of sleep disturbance was significantly higher in the working-from-home group than in the conventional-work group (n = 264, 32.3% vs. n = 253, 6.2%; *P* < 0.001).

**Table 1:** General characteristics of the study population

	Working from home			Conventional work		*P-value*
	N	%		N	%	
	818			4.090		
**Insomnia**						
Difficulty initiating sleep^†^	325	39.7		502	12.3	< 0.0001
Difficulty maintaining sleep^†^	321	39.2		444	10.9	< 0.0001
Non-restorative sleep†	313	38.3		533	13.0	< 0.0001
All three types of sleep disturbance^*†^	264	32.3		253	6.2	< 0.0001
**Age (years), mean ± SD**	47.8 ± 14.1			46.2 ± 13.9		0.002
**Sex**						1.000
Male	279	34.1		1,395	34.1	
Female	539	65.9		2,695	65.9	
**Education**						0.406
≤ High school	438	53.6		2,125	52.0	
≥College	380	46.5		1,965	48.0	
**Status of employment**						0.002
Full-time	625	76.4		3,314	81.0	
Part-time	193	23.6		776	19.0	
**Working period (years), mean ± SD**	5.2 ± 5.8			5.7 ± 6.3		0.031
**Working hour (years), mean ± SD**	39.0 ± 13.0			41.5 ± 12.9		< 0.0001
**Working-home balance**						
3 (Good)	324	36.9		2,473	60.5	
2 (Average)	246	30.1		1,140	27.9	
1 (Bad)	248	30.3		477	11.7	
**Self-perceived health**						0.533
3 (Good)	563	68.8		2,892	70.7	
2 (Average)	230	28.1		1,088	26.6	
1 (Bad)	25	3.1		110	2.7	
**Depression**						< 0.0001
Yes	37	4.5		87	2.1	
No	781	95.5		4,003	97.9	
**Anxiety**						< 0.0001
Yes	40	4.9		104	2.5	
No	778	95.1		3,986	97.5	

^*^All three types of insomnia, difficulty initiating sleep, difficulty maintaining sleep, and non-restorative sleep, simultaneously

^†^Percentage is calculated using the formula (number of people experiencing each type of sleep disturbances/total number of people in each group of workers) × 100

Working from home significantly increased the odds of experiencing all three types of sleep disturbances by 4.79 (95% CI, 3.78–6.07) (Table 2). Furthermore, the odds ratios of each sleep disorder subcategory; “difficulty initiating sleep,” “difficulty maintaining sleep,” and “non-restorative sleep” increased by 3.23 (95% CI, 2.67–3.91), 3.67 (95% CI, 3.02–4.45), and 3.01 times (95% CI, 2.46–3.67), respectively.

**Table 2:** Associations of working from home and insomnia with its sub-items

	Working from home	
	Odds ratio	95% confidence interval
All three types of insomnia*	4.79	3.78–6.07
Difficulty initiating sleep	3.23	2.67–3.91
Difficulty maintaining sleep	3.67	3.02–4.45
Non-restorative sleep	3.01	2.46–3.67

^*^All three types of insomnia, difficulty initiating sleep, difficulty maintaining sleep, and non-restorative sleep, simultaneously

## Discussion

In this study, we determined the relationship between insomnia and working from home compared with conventional work. Our results show that working from home was associated with sleep disturbances, especially insomnia. The proportion of part-time workers was higher and the number of working periods and working hours was shorter in the work-from-home group. Moreover, work-life balance and self-perceived health were worse, and the proportion of people who experienced depression and anxiety was higher.

Among the factors, work-life balance was significantly correlated with working from home and all types of insomnia, implying that a worse work-life balance is paralleled with a higher likelihood of insomnia. Improved familial relationships promote a positive work-life balance; however, as the boundary between family and workspace blurs, it can be difficult to maintain efficient work performance, separate work hours, and focus on family [19]. Thus, whilst working from home has the advantage of promoting a positive work-life balance and allowing for more flexibility, it can also be stressful as it requires workers to perform work and family duties simultaneously in a shared space. As a result, conflicts may occur due to competing demands for professional and familial roles [20].

Anxiety was also significantly correlated with working from home and insomnia. A higher rate of anxiety was observed in the working-from-home group than in the conventional-work group, which is consistent with results from a previous study [21]. Among several factors, it is suggested that people who work from home may feel anxious about not having direct contact with their superiors; thus, not being able to position themselves more prominently in relation to promotion opportunities [22–24]. In this study, there were significantly more part-time workers in the working-from-home group, which might have also contributed to higher levels of anxiety. Part-time workers frequently feel anxious about the possibility of having fewer opportunities to advance their careers compared with full-time workers [25].

Previous studies reported that working from home allows individuals to adjust their sleep cycles for better alignment with their natural circadian rhythm, potentially enhancing sleep quality [26, 27]. Additionally, it was proposed that working from home may also resolve the issue of lack of sleep, which is a chronic problem that plagues modern society [28]. However, irregular life patterns, stress, low physical activity, and psychological factors can interfere with sleep. Moreover, social isolation, which is thought to be the most important disadvantage of working from home, should be considered [29]. Employees who work from home experience loneliness and social isolation due to the absence of social interactions with co-workers [30]. Therefore, if people work from home, real-time communication and information sharing must be maintained with employers and co-workers. People who work from home also had significantly lower physical movement compared with those who worked from the office. Regular exercise, walking while working, and short-term movement to nearby stores or restaurants can maintain a healthy body. The prevalence of cardiometabolic diseases increased during the COVID-19 pandemic due to increased time spent at home [31]. Further, vigorous daytime activities are also closely related to deeper and high-quality sleep [32]. Therefore, the International Labor Organization and the Ministry of Employment and Labor should set work-from-home guidelines and recommend that workers perform regular exercise and take breaks during work, and also familiarize themselves with virtual and video meetings to facilitate communication [33].

This study had several limitations. First, the 5th KWCS was conducted in 2017, pre-COVID-19, which implies that the ratio and diversity of workers working from home may be different from the present one. As various work-related characteristics may undergo changes, further studies comparing the characteristics of workers who work from home before and after the COVID-19 outbreak are important to inform work-from-home guidelines and policies. Second, we matched participants based on sex and occupation only, but other important variables related to sleep, such as age, should also be considered. Future studies evaluating the impact of more complex factors should be conducted. Third, due to the cross-sectional study design, reverse causality could not be entirely excluded (i.e., the possibility that individuals with lower health status and comorbidities, including insomnia, may be more likely to work from home than from the office), and the results should be interpreted cautiously. Further studies employing a longitudinal design are warranted to confirm the hypotheses of this study. Finally, selection bias and the possibility of other conflating variables not accounted for in this study, such as the impact of individual circumstances and occupational characteristics, may be present.

## Conclusion

In conclusion, insomnia is an important topic not only for personal health but also social productivity. Our study findings can be used as a fundamental basis for the implementation of policies and guidelines to prevent insomnia in workers who work from home. Further studies should compare the differences in working-from-home characteristics before, during, and after the COVID-19 outbreak.

## Electronic supplementary material

Below is the link to the electronic supplementary material.

Supplementary Material 1

## Data Availability

The datasets generated and/or analysed during the current study are available in the Korea Occupational Safety and Health Agency website [https://oshri.kosha.or.kr/oshri/researchField/workingEnvironmentSurvey.do].
