# Supplementary material for: Relationship between insomnia and working from home among Korean domestic workers: results from the 5th Korean working condition survey
Source: BMC Public Health. 2023 Jul 17;23:1367. doi: 10.1186/s12889-023-16268-5 (PMC10353197; doi:10.1186/s12889-023-16268-5)
Supplement: Supplementary file 1 — Supplementary Material 1 [file 12889_2023_16268_MOESM1_ESM.docx]

**Supplementary Material**

**Relationship between Insomnia and Working from Home among Korean domestic workers: Results from the 5th Korean Working Condition Survey**

Lei Lee^1†^, Ok Hyung Nam^2,3†^, Ko Eun Lee^3^, and Chunui Lee^4^*^*^*

**Supplementary Methods**

Diagnostic criteria A and C for DSM-V’s definition of sleep disorder and insomnia (ICD disease code G47.00)

A: A predominant complaint of dissatisfaction with sleep quantitiy, associated with on (or more) of the following symptoms:

- Difficulty initiating sleep.
- Difficulty maintaining sleep, characterized by frequent awakenings or problems returning to sleep after awakenings.
- Early-morning awakening with inability to return to sleep.

C: The sleep difficulty occurs at least 3 nights per week.
